# Correlation in endophytic fungi community diversity and bioactive compounds of *Sophora alopecuroides*

**DOI:** 10.3389/fmicb.2022.955647

**Published:** 2022-08-31

**Authors:** Mingxiu Ju, Qingchen Zhang, Ruotong Wang, Siyuan Yan, Zhengnan Li, Peng Li, Peiwen Gu

**Affiliations:** ^1^School of Agriculture, Ningxia University, Yinchuan, China; ^2^Department of Pharmacotherapy and Translational Research, University of Florida, Gainesville, FL, United States; ^3^College of Horticulture and Plant Protection, Inner Mongolia Agricultural University, Hohhot, China; ^4^State Key Laboratory of High-Efficiency Utilization of Coal and Green Chemical Engineering, Ningxia University, Yinchuan, China

**Keywords:** *Sophora alopecuroides*, fungal endophyte, quinolizidine alkaloids, high-throughput sequencing, medicinal plant

## Abstract

*Sophora alopecuroides* L. is a traditional Chinese medicine used for the treatment of several different disease states including bacillary dysentery and enteritis. But importantly, it also plays a role as an anti-tumor agent. That said, little is known about the role endophytes play regarding the clinically bioactive metabolites in *S. alopecuroides*. In order to explore the effects of endophytic fungi on the accumulation, quality, and correlation in the content of the medicinal compounds, the structural diversity of endophytic fungi in *S. alopecuroides* was analyzed. The relationship between endophytes and quinolizidine alkaloids (QAs), housed within the seeds of *S. alopecuroides*, which were interpreted based on established methods of high-throughput sequencing and high-performance liquid chromatography. A total of 1,034,418 effective sequence reads and 257 operational taxonomic units (OTUs) were obtained from 33 samples which were sourced from 11 different sampling sites and further classified into 9 phyla, 20 classes, 45 orders, 85 families, and 118 genera. Ascomycota was found to be the dominant phylum of endophytic fungi in *S. alopecuroides*, with a relative abundance ranging from 60.85 to 98.30%. *Alternaria*, *Cladosporium*, *Filobasidium*, and an unidentified Ascomycota were the core-shared endophytes, accounting for 49.96, 27.12, 14.83, and 7.88%, respectively. Correlation analysis showed that the Simpson’s diversity index of endophytic fungal community in *S. alopecuroides* was significantly positively correlated with the Oxymatrine (OMA) content in different areas, while the Chao and Shannoneven indexes were significantly negatively correlated with OMA. The endophytic fungi of *Alternaria* were positively correlated with the content of OMA, Oxysophocarpine (OSC), and total QAs. This study has mastered the endophytic fungi resources of *S. alopecuroides*, explored potential functional endophytic fungi, and provided a scientific basis for using biological fertilization strategies to improve the quality of *S. alopecuroides*.

## Introduction

**FIGURE 1:**
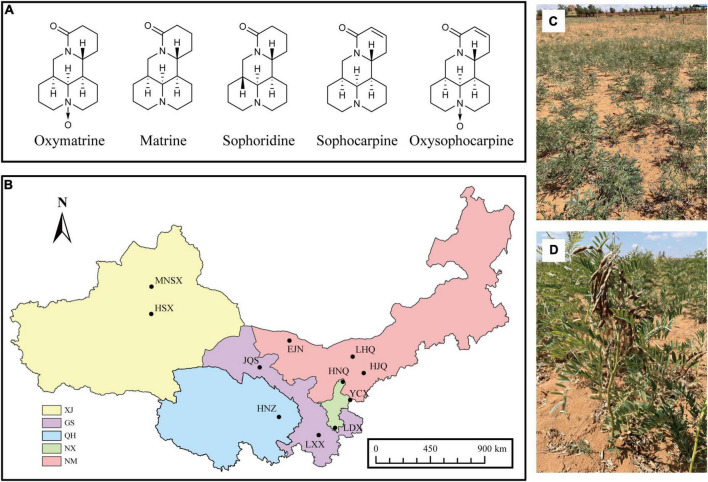
**(A)** Chemical structures of the five quinolizidine alkaloids from *S. alopecuroides*, **(B)** distribution map of sampling plot of *S. alopecuroides*. XJ, Xinjiang Autonomous Region, GS, Gansu Province, QH, Qinghai Province, NX, Ningxia Autonomous Region, NM, Inner Mongolia Autonomous Region. **(C,D)** Habitat and plants of *S. alopecuroides* (photographed in Yanchi County, Ningxia, China).

*Sophora alopecuroides* L. belongs to perennial Fabaceae herbs, which are mainly distributed in arid and semi-arid areas of the Asian continent, and mainly grows in Northwest China, in regions like Xinjiang, Inner Mongolia, Qinghai, Gansu, Ningxia, and other areas in China (Wang et al., 2020). *S. alopecuroides* has a well-developed root system and is well-suited to tolerate drought, high levels of salinity, and barren conditions. Moreover, it plays an essential role in sand fixation and is an important part of the vegetation in the desert environmental protection of China (Wang et al., 2019). As a traditional Chinese medicine, all tissues of *S. alopecuroides* can be used to treat a variety of different ailments, including reducing fever, relieving pain, and reducing inflammation (Wang et al., 2020). Quinolizidine alkaloids (QAs) are the primary medicinally bioactive compounds of *S. alopecuroides*, which include Oxymatrine (OMA), Matrine (MA), Sophoridine (SR), Sophocarpine (SC), and Oxysophocarpine (OSC) (Zhao et al., 2010; Wang et al., 2018b; Li et al., 2020b; Figure 1A). Artificial cultivation of *S. alopecuroides* is uneconomical as there are problems with low germination rates, slow growth, high costs, and low yield of the bioactive compounds. This makes it difficult to meet the large demand for *S. alopecuroides* in the medicinal market (Song et al., 2018; Zhang J. G. et al., 2020). The barriers of viable artificial cultivation options have led to excessive harvesting and the subsequent degradation of grasslands and destruction of the ecological balance in associated regions (Wang et al., 2019). Therefore, understanding the biological characteristics of *S. alopecuroides* and improving the traditional cultivation methods in a scientific way are important measures to improve the quality of its medicinal materials.

Previous studies have shown that the bioactive compounds of medicinal plants are influenced by several different environmental factors including temperature, rainfall, and light. Furthermore, there are just as many internal factors to be taken into consideration for instance endophytes, genetics, and enzymes (Li et al., 2020a; Pang et al., 2021). It is worth noting that endophytic fungi not only regulate the synthesis and accumulation of medicinally bioactive compounds of their hosts but also produce secondary metabolites similar to that of their host plants, which are potential raw materials for medicine (Pang et al., 2021). However, the relationship between endophytic fungi of medicinal plants and the content of medicinal compounds in the host, as well as the mechanism of endophytic fungi promoting the accumulation of bioactive compounds in medicinal plants are still unclear.

A large number of studies have shown that endophytes are symbiotic fungi that systematically colonize, without inducing a host response, and interact to form a relatively stable micro-ecosystem with the host (Hassani et al., 2018). The function of endophytic fungi for the accumulation of bioactive compounds in the host is the most important part of research on the biological characteristics of medicinal plants. The impact of endophytic fungi on medicinal plants can be considered from at least three aspects: (1) The endophytes can produce nitrogen, phosphorus, and growth hormones such as gibberellins, indole acetic acid, and ethylene in the host, promote host growth and accumulation of secondary metabolites, and help plants cope with biotic and abiotic stresses (Rodriguez et al., 2009; Chen et al., 2020; Morella et al., 2020; Daroodi et al., 2021). (2) Endophytic fungi could act as elicitors to induce the expression of host secondary metabolism-related genes and the subsequent production of new metabolites (Li et al., 2019; de Amorim et al., 2020). (3) Biotransformation in endophytic fungal metabolites and host metabolites alters plant metabolite composition (Tian et al., 2014; Maggini et al., 2017; Ekiz et al., 2019). However, the relationship between endophytic fungi of *S. alopecuroides* and the bioactive compounds of the host has yet to be reported.

Compared with traditional research methods such as microscopic observation, separation and culture, and denaturing gradient gel electrophoresis, high-throughput sequencing has the advantages of generating a large amount of information and good parallelism between samples, which gives a more comprehensive reflection of the structure of the microbial community in samples. For these reasons, this technique has been widely used in the research of plant endophytes and environmental microorganisms (Nilsson et al., 2019). Therefore, this study used the MiSeq sequencing platform and combined bioinformatics to analyze the endophytic fungal community structure and diversity of *S. alopecuroides* seeds and to grasp the overall status of the endophytic fungal resources. At the same time, the content of bioactive compounds housed within *S. alopecuroides* seed samples from different regions was determined and the correlation between endophytic fungi and the bioactive compounds of the host was analyzed by multivariate correlation. Our study provides a scientific basis for finding endophytic fungal resources that can increase the content of bioactive compounds, and the utilization of biological fertilization strategies to improve the quality of *S. alopecuroides*.

## Materials and methods

### Experimental materials

In September 2020, mature legumes from wild *S. alopecuroides* were collected from 11 wild *S. alopecuroides* distribution areas in northwest China (Figure 1B and Supplementary Table 1), morphologically identified as *Sophora alopecuroides* L. (Figures 1C,D). Each sampling plot was set as a “Z”-shaped sampling point and had a total area of 25 m^2^. A total of 500 legumes were randomly collected from 3 sampling points in each plot, providing a total of 1,500 legumes. A total of 33 samples from 11 sampling plots were brought back to the laboratory for threshing. Pests and samples that were withered and mechanically damaged were removed from each sample. The samples were further prepared by using the quartering method which left approximately 5 g of seeds per sample. The seeds were then soaked in 75% ethanol for 30 s and then in sodium hypochlorite, with an available chlorine content of 1%, for 3 min. The seeds were surface sterilized under aseptic conditions, rinsed with sterile water 3 times and drained through filter paper. To confirm the successful disinfection of the seed surface, the final water rinse was spread on a potato dextrose agar (PDA) plate and cultured for 3 days in the dark at 25°C. If no colonies were observed, the seeds were assumed to be adequately disinfected and were used for subsequent experiments. The sterilized seeds were equally divided into two parts, one was stored in a sterile tube for high-throughput sequencing of endophyte fungi of *S. alopecuroides* and the other was pulverized to a homogeneous powder by a freeze-drying grinder (100 mesh) for the extraction and determination of the bioactive compounds. All samples were stored frozen at −80°C in an ultra-low temperature freezer.

The reference standards required for the determination of bioactive compounds in *S. alopecuroides*, including OMA, MA, SR, SC, and OSC, were purchased from National Institutes for Food and Drug Control.

### Extraction and content determination of quinolizidine alkaloids from *Sophora alopecuroides*

The extraction method was modified according to the research of Wang et al. (2018a). A total of 0.25 g of dried powdered seeds was placed into a 50 mL conical flask containing 0.08 g cellulase (10 U/g). Next 40 mL of methanol was added to the conical and vortex to mix for 10 s. The mixture was sonicated at 45°C for 60 min and then centrifuged at 13,000 rpm for 10 min. The supernatant extract was concentrated by removing methanol using a rotary vacuum evaporator at 50 °C and dissolved in 10 mL mobile phase. The solution was passed through a 0.45-μm filter membrane for detection by quantitative and qualitative analysis.

HPLC analysis was performed using Agilent 1260 Infinity II system (Agilent Technologies, Santa Clara, CA, United States). The separation of different bioactive compounds was on a reverse-phase column (ZORBAX SB-C18 column, 4.6 × 250 mm, 5 μm, Agilent, United States). Chromatographic conditions: mobile phase 0.2% phosphoric acid (triethylamine 2.00 mol/L) (A) and acetonitrile (B), elution gradient 0∼22 min, 96.5%A; 22∼23 min, 96.5∼0%A; 23∼27 min, 100%A, 27∼28 min, 0∼96.5%A; 28∼34 min, 96.5%A, detection wavelength 205 nm, flow rate 1.0 mL/min, injection volume 10 μL, and column temperature 30°C.

Preparation of reference standard solution: Each reference standard was prepared by adding a total of 10 mg of either OMA, MA, OSC, or SC reference standard to a volume of 1,000 μL of the mobile phase. Each sample was then semi-sequentially diluted to appropriate concentrations with mobile phase, assayed under the chromatographic conditions described above and a standard curve was drawn (Supplementary Table 2). Qualitative analysis was performed according to the retention times of authentic standards. Quantitative measurement was conducted by comparing the detection results to standard curves that had been constructed according to concentrations and peak areas of reference standards in the chromatograms.

Mass spectrometry detection according to the method of Wang et al. (2018b) with some modifications. LC-MS analysis was performed on an Agilent 1290II-G6546A UPLC-ESI-Q-ToF mass spectrometer (Agilent Technologies, Santa Clara, CA, United States). Samples and standard alkaloids were detected by positive ionization mode with a capillary voltage of 3,000 V and a fragmentor voltage of 170 V. The mass spectra were recorded from m/z 100 to 1,000. Then, the mass spectrum of individual peaks in the total ion chromatography of the extracting solution of *S. alopecuroides* seeds was compared with the mass spectrum of authentic standards of OMA, MA, OSC, SC, and SR to verify the existence of these alkaloids.

### Total DNA extraction and high-throughput sequencing of *Sophora alopecuroides*

To characterize the endophytic fungal community in *S. alopecuroides* in different areas, genomic DNA was extracted from the sterilized seeds of *S. alopecuroides* by use of a Fast DNA SPIN kit (MP Biomedicals, Santa Ana, CA, United States). The quality of DNA was detected by 1% agarose gel electrophoresis, and the concentration and purity of DNA were determined by NanoDrop 2000. The fungal internal transcribed spacer (ITS rDNA) was amplified using ITS1F (5′-CTTGGTCATTTAGAGGAAGTAA-3′) and ITS2R (5′-GCTGCGTTCTTCATCGATGC-3′). Amplification, purification, and sequencing library construction were referenced in Zhang X. G. et al. (2020). Purified amplicons were pooled in equimolar and paired-end sequenced (2 × 300) on an Illumina MiSeq platform (Illumina, San Diego, United States) according to the standard protocols by Majorbio Bio-Pharm Technology Co., Ltd. (Shanghai, China). The raw reads were deposited into the NCBI Sequence Read Archive (SRA) database (Accession Number: SRP373295).

### Bioinformatics and statistical analysis

The paired-end reads from sequencing were assigned to respective samples using Cutadapt software (Martin, 2011), based on their specificity. Barcode and primer sequences were truncated from these reads. Flash software version 1.2.7 (Magoc and Salzberg, 2011) was employed to cut and splice the remaining paired-end reads of each sample, and these reads were assembled to obtain the original label. High-quality clean data was obtained by filtering the original data under specific filtering conditions (Caporaso et al., 2010; Bokulich et al., 2013). Additionally, to segregate chimeric sequences and to eliminate the non-microbial reads, for instance, chloroplast and mitochondrial reads, the reads were compared with the Unite database^1^ (Alawneh et al., 2006) using UCHIME (Edgar et al., 2011). Thus, clean reads were generated. UPARSE software version 7.0.1001^2^ (Edgar, 2013) was used to cluster the sequences into the same operational taxonomic units (OTUs) with ≥ 97% similarity, and a representative sequence with the highest frequency was selected for further annotation. The Unite database (Release 8.0) see footnote 1 was used to execute annotated information for each representative sequence. OTUs abundance was normalized using the samples containing the least number of sequences. Subsequent analyses were based on the normalized data.

In this study, the alpha diversity index, including Shannoneven (Reflecting community uniformity), Simpson (Reflecting community diversity), Chao (Reflecting community richness), and PD (Reflecting the pedigree diversity of species) of the samples were calculated using the QIIME version 1.7.0 and represented using R software version 2.15.3. The average values of relative abundance of each group were used to construct species histograms of phylum, class order, and genus. R software version 2.15.3 was employed to perform the principal coordinate analysis (PCoA) based on Bray-Curtis, ANOSIM, and Venn diagram. The statistical analysis of variance (ANOVA), Kruskal–Wallis rank sum test, and Spearman correlation analysis were performed using SPSS version 19.0 (IBM Inc., Armonk, NY, United States), with the significance level set to 0.05, 0.01, and 0.001.

## Results

### Diversity of endophytic fungi in *Sophora alopecuroides* in different areas

A total of 1,927,670 high-quality sequences were obtained by high-throughput sequencing of endophytic fungi in *S. alopecuroides* seeds from 11 sampling areas. To minimize the effects of sequencing depth on alpha and beta diversity measures, the number of reads from each sample was rarefied to 31,346. Finally, 1,034,418 effective reads (33 samples) were obtained and grouped into 257 OTUs, based on a threshold of 97% sequence similarity. The rarefaction curve reflects the sequencing depth of the sample, which can be used to assess whether the sequencing volume is sufficient to cover all taxa. With the increase of sequencing depth, the rarefaction curve of OTU number in the 33 samples tends to be stable, which indicated that the amount of sequencing data was gradually becoming reasonable (Supplementary Figure 1).

The α diversity of endophytic fungi of *S. alopecuroides* in the 11 sampling areas was assessed by the number of sample sequences, community diversity (Simpson index), evenness (Shannoneven index), richness (Chao index), and lineage diversity of species (PD index) (Table 1). The sample area with the largest number of sequences detected in each sample was JQS, with 69,769 sequences, and the sample area with the least number was HNZ, with 34,487 sequences. The Shannoneven index of endophytic fungi of *S. alopecuroides* in the plots HNZ and MNSX were the highest, both 0.51, which were significantly higher than those in the plots HNQ and YCX (0.21 and 0.17). While the Simpson index was quite the opposite, with significantly higher endophytic fungi in HNQ and YCX (0.77 and 0.82) than in HNZ and MNSX (0.31 and 0.33). This result indicates that the diversity and evenness of endophytic fungi presented differences among the four plots of *S. alopecuroides*. The Chao index of HNZ was the highest at 21.26, followed by YCX at 20.33, and the lowest among all samples was EJN and HSX, which were both 12.67. The highest PD index was YCX (6.82), followed by HNZ (6.61) and the lowest was EJN (4.63). The richness and lineage diversity of endophytic fungi in *S. alopecuroides* were similar among all samples of the 11 plots.

**TABLE 1:** Alpha diversity index of fungal community in *S. alopecuroides* seeds.

Sample	Mean ± LSD (*n* = 3)				
	
	Sequence	Shannoneven	Simpson	Chao	pd
EJN	66,498 ± 3,273	0.46 ± 0.09	0.43 ± 0.11	12.67 ± 1.2	4.63 ± 0.15
HJQ	60,422 ± 2,495	0.49 ± 0.06	0.34 ± 0.06	16.67 ± 2.33	6.17 ± 0.63
HNQ	51,821 ± 3,804	**0.21 ± 0.05b**	**0.77 ± 0.06a**	14.00 ± 1.73	5.19 ± 0.64
HNZ	34,487 ± 2,497	**0.51 ± 0.05a**	**0.31 ± 0.07b**	21.67 ± 3.76	6.61 ± 1.03
HSX	68,355 ± 2,191	0.38 ± 0.05	0.56 ± 0.06	12.67 ± 0.67	4.86 ± 0.27
JQS	69,769 ± 2,270	0.26 ± 0.08	0.66 ± 0.12	14.00 ± 1.16	5.51 ± 0.33
LDX	50,534 ± 9,853	0.37 ± 0.06	0.55 ± 0.08	17.33 ± 2.96	6.29 ± 0.58
LHQ	64,134 ± 5,765	0.40 ± 0.09	0.5 ± 0.14	14.67 ± 3.84	5.61 ± 1.05
LXX	62,076 ± 5,539	0.40 ± 0.11	0.49 ± 0.16	17 ± 6.03	6.23 ± 1.79
MNSX	65,071 ± 6,845	**0.51 ± 0.07a**	**0.33 ± 0.07b**	17.78 ± 1.31	6.47 ± 0.21
YCX	49,390 ± 2,326	**0.17 ± 0.04b**	**0.82 ± 0.05a**	20.33 ± 1.2	6.82 ± 0.12

The bold values and different letters (a and b) indicate significance at *p* = 0.05 level.

**FIGURE 2:**
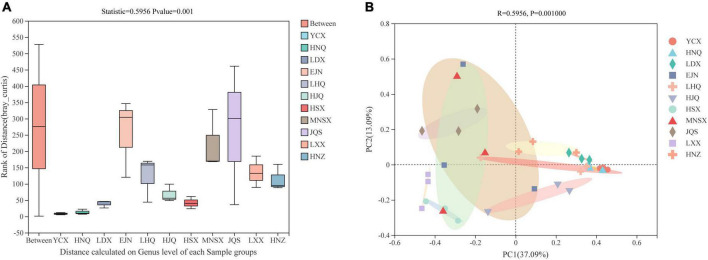
Similarity ANOSIM analysis and PCoA analysis of endophytic fungal community in *S. alopecuroides* based on Bray-Curtis distance. **(A)** The *X*-axis is the distance value within or between groups, the box corresponding to “Between” represents the distance value of the difference between the groups, and the rest of the boxes represent the distance value of the difference within the group; the *Y*-axis scale represents the size of the distance value. **(B)** The *X*-axis and *Y*-axis represent the two selected main coordinate axes, and the percentage represents the interpretation value of the main coordinate axis for the difference in sample composition; The scales of the *X*-axis and *Y*-axis are relative distances and have no practical significance; Points with different colors or shapes represent different groups of samples, and the closer the two sample points are, the more similar the species composition of the two samples is.

In the ANOSIM analysis based on the Bray–Curtis distance algorithm (Figure 2A), Bray-Curtis clustering was performed on the endophytic fungal community of *S. alopecuroides* at 11 sampling plots (Statistic = 0.5799), and the difference between sample groups was significantly greater than that within the group (*P* = 0.001), which indicates that the selection of sampling points in this experiment was statistically significant. The PCoA, based on the Bray–Curtis distance algorithm, showed that there were certain differences in the endophytic fungal community of *S. alopecuroides* in different plots (*R* = 0.5799, *P* = 0.001) (Figure 2B). The PC1 axis explained 36.73% of the data and the PC2 axis explained 13.29%. The endophytic fungal community of *S. alopecuroides* in 11 plots was divided into two groups. The samples in the YCX, HNQ, LDX, LHQ, HJQ, and HNZ plots were relatively compact, which have relatively similar endophytic fungal communities, while the samples in the HSX, MNSX, JQS, LXX, and EJN plots were relatively loose and were greatly varied in the diversity of endophytic fungal communities.

### Composition of endophytic fungal community in *Sophora alopecuroides*

**FIGURE 3:**
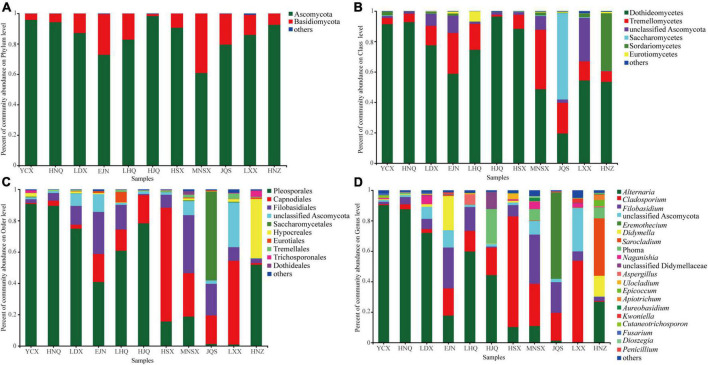
Composition of endophytic fungal community of *S. alopecuroides* in different areas. **(A)** Phylum level, **(B)** class level, **(C)** order level, and **(D)** genus level.

The endophytic fungal community composition of *S. alopecuroides* in 11 plots was divided into 9 phyla, 20 classes, 45 orders, 85 families, and 118 genera. The endophytic fungi of *S. alopecuroides* seeds with a relative abundance of less than 0.01% at each classification level were merged. Figure 3 shows that the dominant phyla of *S. alopecuroides* endophytes were Ascomycota among all 11 sample areas, with a relative abundance from 60.85 to 98.30% (Figure 3A). At the class level (Figure 3B), the dominant class of endophytic fungi of *S. alopecuroides* is Dothideomycetes, the relative richness ranged from 48.67 to 96.37%, except for JQS whose dominant class is Saccharomycetes with the relative abundance of 56.78%. At the order level (Figure 3C), the dominant order of endophytic fungi in HSX and LXX *S. alopecuroides* was Capnodiales, with relative abundances of 72.61 and 53.43%, respectively. The dominant order of the endophytic fungi of *S. alopecuroides* in MNSX was Filobasidiales, with a relative abundance of 37.03%. The dominant orders of the endophytes of *S. alopecuroides* in other plots were Pleosporales, with a relative abundance ranging from 40.87 to 90.71%. At the genus level (Figure 3D), the dominant genus of endophytic fungi in YCX, HNQ, LDX, LHQ, and HJQ is *Alternaria*, with relative richness of 90.21, 87.55, 71.20, 59.78, and 44.29%, respectively. The dominant genus of endophytic fungi of *S. alopecuroides* in HSX and LXX was *Cladosporium*, with relative abundances of 72.61 and 53.43%, respectively. The dominant genus of endophytic fungi of *S. alopecuroides* in EJN and MNSX was *Filobasidium*, with relative abundances of 26.81 and 32.31%, respectively. The dominant genus of endophytic fungi of *S. alopecuroides* in JQS was *Eremothecium*, with a relative abundance of 56.78%. The dominant genus of endophytic fungi of *S. alopecuroides* in HNZ was *Sarocladium*, with a relative abundance of 37.71%.

**FIGURE 4:**
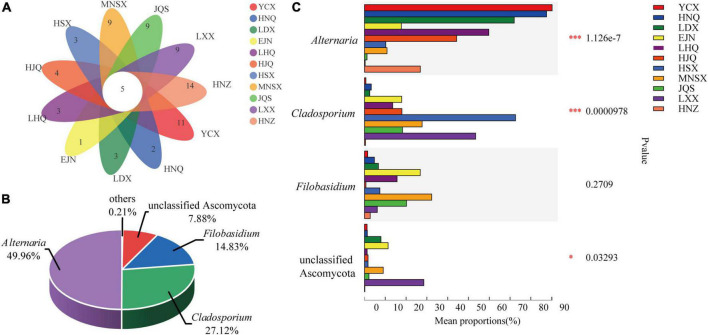
Species composition and difference test of endophytic fungal community in *S. alopecuroides.* **(A)** Venn plot based on genus level representing common or unique genera in the given population. **(B)** The percentage of core-shared genera. **(C)** Difference test between groups, the *Y*-axis represents the species name under a certain taxonomic level, the *X*-axis represents the average relative abundance of species in different groups, and the columns with different colors represent different groups. The far right is the *P*-value (one-way ANOVA, *P < 0.05, ***P < 0.001).

The Venn diagram shows the number of individual and shared endophytic fungal genera in *S. alopecuroides* endophyte community among 11 areas (Figure 4A). There are 5 core-shared genera of endophytic fungi in *S. alopecuroides*, including *Alternaria*, *Cladosporium*, *Filobasidium*, unclassified Ascomycota, and unclassified fungi (Supplementary Table 3). Further analysis of the relative richness of the core-shared genera of *S. alopecuroides* endophytes (combining less than 1% of the core-shared genera as others) can be found in Figure 4B. *Alternaria* was the most core-shared genera, accounting for 49.96%, followed by *Cladosporium*, accounting for 27.12% of the core-shared genera. The significant differences between groups of core-shared genera were analyzed for the endophytic fungi of *S. alopecuroides* in the 11 plots (one-way ANOVA) (Figure 4C). The results showed that *Alteinaria* and *Cladosporium* had extremely significant differences (*P* < 0.01) among the samples of *S. alopecuroides* in the 11 plots. Unclassified Ascomycota were significantly different among *S. alopecuroides* samples in each plot (*P* < 0.05). The phylogenetic tree was constructed by species annotation and artificial Blast comparison, which was rooted to *Trypethelium* sp. (DQ782839) (Huang et al., 2009). The *Alternaria* was identified as *Alternaria alternata*, Cladosporium could not be identified as a species, the *Filobasidium* was identified as *Filobasidium magnum*, and the unclassified Ascomycota may be an endemic endophyte to *Sophora* sp. (Supplementary Figure 2).

### Determination of bioactive compounds content of *Sophora alopecuroides* seeds in different areas

**FIGURE 5:**
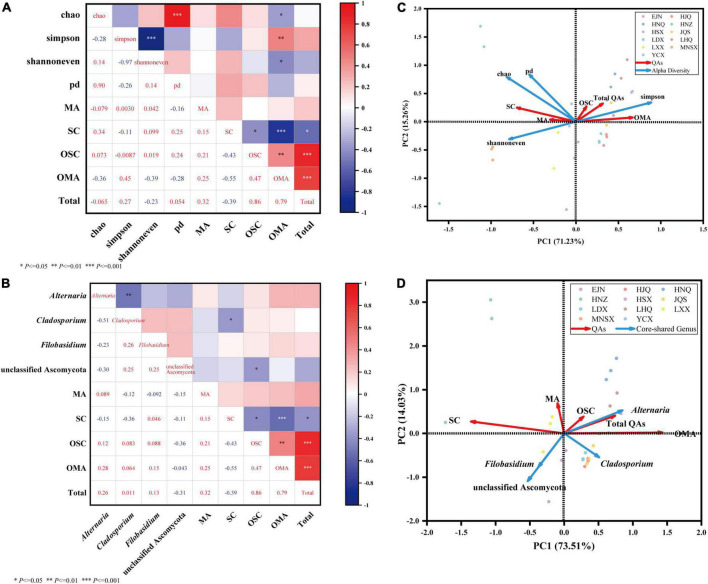
Community diversity of endophytic fungi in seeds of *S. alopecuroides* and correlation analysis of core-shared genera and bioactive compounds. Sections **(A,C)** are heatmaps of Spearman correlation analysis of endophytic fungal diversity and core-shared genera and host bioactive compounds, respectively. * Means *P* < 0.05, ** means *P* < 0.01, *** means *P* < 0.001. Sections **(B,D)** are the redundancy analysis of endophytic fungal diversity and host bioactive compounds, respectively, blue represents the alpha diversity index and core genus fungi, red represents four bioactive compounds, different colored dots represent samples, and sharp angles represent positive correlations, the obtuse angle indicates negative correlation.

The HPLC detection results of the bioactive compounds of the standards and samples are shown in Supplementary Figures 3A,B. It was indicated that the five QAs in *S. alopecuroides* seeds can be effectively detected under this HPLC condition. The individual mass spectrum verified the existence of these bioactive compounds (Supplementary Figures 3C–L). The SC in the analysis results represents the total content of SC and SR because their retention time of them had little difference. Difference analysis by the Kruskal-Wallis rank sum test showed that there were extremely significant differences (*P* < 0.01) in the content of bioactive compounds in *S. alopecuroides* seeds in different areas (Table 2). From the perspective of total alkaloids of QAs, the content of LHQ in the sample area was the highest at 486.85 mg/g, followed by the sample area of HNQ, at 483.84 mg/g, and the least in the sample area of MNSX, at 329.34 mg/g. From the perspective of a single alkaloid, the OMA content from the HNQ sample area was the highest at 238.90 mg/g, followed by HSX at 224.89 mg/g, and the least in MNSX at 89.27 mg/g. The content of OSC in HNZ was the highest at 216.47 mg/g, followed by LHQ at 211.01 mg/g, and the least in EJN at 122.42 mg/g. The main QAs alkaloids in *S. alopecuroides* seeds are OSC and OMA, accounting for 32.96∼48.24% and 27.11∼49.38% of the total QAs, respectively. Followed by SC which accounts for 10.10–31.30% of the total QAs, and the least content is MA, which only accounts for 0.30–1.60%. Based on Spearman correlation heatmaps in Figures 5A,B, there is an extremely positive correlation between OMA, OSC, and the content of the total QAs in *S. alopecuroides* (*p* < 0.001), and the correlation coefficients were 0.79 and 0.86. This indicates that the higher the content of OMA and OSC, the higher content of the total QAs. Therefore, subsequent studies will focus on the correlation between content of OMA, OSC, and the total QAs and the endophytic fungi of *S. alopecuroides*.

**TABLE 2:** Content of QAs in *S. alopecuroides* seeds.

Sample	Mean ± LSD (*n* = 3), (mg/g)				
	
	MA	SC	OSC	OMA	Total QAs
EJN	3.20 ± 0.21	63.78 ± 3.91	122.42 ± 7.41	182.00 ± 11.26	371.41 ± 22.79
HJQ	2.30 ± 0.02	47.58 ± 0.40	193.20 ± 1.83	177.61 ± 1.71	420.69 ± 3.90
HNQ	6.00 ± 0.09	57.84 ± 0.80	181.10 ± 3.71	238.90 ± 4.55	483.84 ± 9.15
HNZ	7.25 ± 0.11	112.62 ± 2.01	216.47 ± 3.11	112.44 ± 1.43	448.79 ± 5.34
HSX	3.56 ± 0.06	48.01 ± 1.04	198.97 ± 5.24	224.89 ± 6.09	475.44 ± 2.40
JQS	1.97 ± 0.07	57.26 ± 1.07	188.13 ± 3.40	197.29 ± 3.30	444.65 ± 7.83
LDX	2.75 ± 0.03	55.11 ± 0.39	167.24 ± 1.29	193.07 ± 1.44	418.17 ± 3.14
LHQ	3.39 ± 0.11	54.86 ± 2.33	211.01 ± 8.03	217.59 ± 8.51	486.85 ± 18.96
LXX	4.27 ± 0.12	69.64 ± 2.90	153.19 ± 5.89	164.07 ± 6.55	391.19 ± 15.47
MNSX	1.00 ± 0.02	103.10 ± 1.14	135.97 ± 1.82	89.27 ± 0.96	329.34 ± 3.84
YCX	2.45 ± 0.08	77.88 ± 1.38	158.89 ± 2.49	181.27 ± 3.13	420.49 ± 7.03

### Correlation analysis of endophytic fungi in *Sophora alopecuroides* and their bioactive compounds

Figure 5A the Spearman-based correlation heat map shows that the diversity (Simpson index), evenness (Shannoneven index), richness (Chao index), and lineage diversity of species (PD index) of the endophytic fungal community in *S. alopecuroides* were significantly correlated with the content of bioactive compounds. The Simpson index was positively correlated with OMA (*P* < 0.01), and the correlation coefficient was 0.45, which indicates that the greater the Simpson index, the simpler the endophytic fungal community composition, and the higher the OMA content. The Chao index and Shannoneven index were significantly (*P* < 0.05) negatively correlated with OMA, the correlation coefficients were −0.36 and −0.39, respectively. This indicates that the greater the Chao index and the Shannoneven index, the richer the endophytic fungal community composition and the lower the OMA content. Data response measures were evaluated using Canoco 5, and Redundancy analysis (RDA) was recommended to evaluate the correlation between endophytic fungi and bioactive compounds (Figure 5C). The results showed that the endophytic fungal community Simpson index was positively correlated with the total amount of OMA, OSC, and QAs, while other indices were negatively correlated, which was consistent with the heatmap results.

The relationship between the core-shared genera and effective compounds of the endophytic fungal community of *S. alopecuroides* in the 11 plots was further analyzed. The correlation heatmap in Figure 5B shows that the endophytic fungus *Alternaria* of *S. alopecuroides* is positively correlated with the amount of OMA, OSC, and the total QAs, with the evident correlation coefficients of 0.28, 0.12, and 0.26, respectively. The unclassified Ascomycota of *S. alopecuroides* endophyte was significantly negatively correlated with OSC, and the correlation coefficient was −0.36. In the RDA analysis (Figure 5D), the endophyte *Alternaria* was positively correlated with the amount of OMA, OSC, and the total QAs. It indicated that the endophytic fungus *Alternaria* of *S. alopecuroides* played an important role in the synthesis and accumulation of bioactive compounds of *S. alopecuroides.*

## Discussion

Recent studies have highlighted the importance of endophyte communities on the production of secondary plant metabolites (Pang et al., 2021). Studies have shown that there is an extremely positive correlation between secondary metabolite accumulation and endophytic fungal communities (Pang et al., 2021). The colonization of the endophyte *Epichloë* directly determines whether the host produces ergot alkaloids (Lin et al., 2019). Similarly, the endophyte *Undifilum* or *Alternaria Oxytropis* is directly associated with the host production of indolizidine alkaloids (Cook et al., 2014). In this study, we found that community diversity, evenness, and richness were negatively correlated with the total amount of OMA, OSC, and QAs and that there is a strong positive correlation between *Alternaria* and the total amount of OMA, OSC, and QAs. We speculate that *Alternaria* may produce QAs, which provides new ideas for the mining of endophytic fungal functional strains of *S. alopecuroides*, the breeding of medicinal plants, and the improvement of quality by using biological fertilization strategies.

In this study, MiSeq high-throughput technology was used to sequence the ITS rDNA gene of *S. alopecuroides* endophyte from 33 seed samples from 11 *S. alopecuroides* distribution plots in natural grasslands in northwest China, which objectively and comprehensively reflected the endophytic community structure and diversity of *S. alopecuroides*. We found that the richness and lineage diversity of endophytic fungi in *S. alopecuroides* were similar among the 11 plots, and the diversity and evenness of endophytic fungi of *S. alopecuroides* in the plots HNQ and YCX were significantly different from those in HNZ and MNSX (Table 1). The endophytic fungal community of *S. alopecuroides* in 11 areas was divided into two groups. The YCX, HNQ, LDX, LHQ, HJQ, and HNZ plots have similar endophytic fungal communities in *S. alopecuroides*, which were geographically concentrated and have similar climatic and topographical features. However, the endophytic fungal communities of *S. alopecuroides* in the five plots of HSX, MNSX, JQS, LXX, and EJN were quite different, which was presumed to be related to the complex climate, environment, and topography of these areas (Figure 2). Cui et al. (2019) showed that endophytic fungi are highly related geographically, which is similar to the findings of this study. Several studies have shown a long-term association and co-evolution between some seed-related microorganisms and their hosts (Rybakova et al., 2017; Chen et al., 2018). Chen et al. (2021) analyzed the external factors affecting endophytes in *Rheum palmatum* and found that spatial distance had a greater impact on the community structure of bacterial and fungi endophytes than climate. The interactions between plants and microorganisms are highly dynamic, based on the influence of co-evolutionary and environmental factors, which produce strong deterministic selection effects on microbial communities (Stegen et al., 2012).

It is critical for researchers to study plant species, organs, and the specificity of endophytic fungi to elucidate the effects of endophytic fungi on plant secondary metabolism. Not all endophytes can exhibit specificity to their hosts, only a small percentage of endophytes can exhibit specificity through vertical transmission which is the process of endophytic fungi transmission from parent to progeny, with seeds being the initial inoculum of plant endophytes (Links et al., 2014). The seed microbiome involves a narrow range of microbial species that have evolved through co-selection with their hosts, which provides specific conditions for plant growth and metabolism (Truyens et al., 2013; Samreen et al., 2021). We found that *S. alopecuroides* has selected specific microbial community compositions, mainly Ascomycota, during long-term evolution (Figure 3A). The Ascomycota are highly adaptive and are abundant and diverse in both terrestrial and coastal marine habitat ecosystems (Picard, 2017; Dang et al., 2021). *Alteinaria, Cladosporium*, *Filobasidium*, *Eremothecium*, and *Sarocladium* were the dominant genera in the 11 plots (Figures 3D, 4). *Alternaria*, *Cladosporium*, and *Filobasidium* are common endophytic fungi that enter the next generation through vertical transmission (Nelson, 2017). *Alternaria* is the core microorganism of many seeds, such as corn, rice, and many cruciferous and leguminous plants, which forms a dynamic equilibrium relationship with host plants in long-term co-evolution (Rodriguez et al., 2009). However, once this balance is broken, *Alternaria* will become pathogenic and subsequently damage their hosts (Links et al., 2014; Klaedtke et al., 2016; Chen et al., 2018). For example, *Alternaria solani* can cause early blight of potatoes (Gao et al., 2017). However, endophytic *Alternaria* endows the host with the ability to produce special secondary metabolites (Pang et al., 2021). In some medicinal plants, endophytic *Alternaria* can produce a variety of secondary metabolites, which are important medicinal compounds used in production and in clinics (El-Sayed, 2021; Tian et al., 2021). For example, the secondary metabolites of *A. Oxytropis* include swainsonine (SW), eburicol, and lanosterol, which are all potential medicinal compounds (Song et al., 2019). Lu et al. (2021) conducted a detailed analysis of the metabolic pathway of *A. Oxytropis*, and speculated that the biosynthetic pathway of SW in fungi includes two branches, P6C and P2C, and Saccharopine reductase is involved in the synthesis. In addition, 1-indolizidineone is the direct precursor for the synthesis of SW, and hydroxymethylglutaryl-CoA lyase catalyzes the synthesis of SW (Li and Lu, 2019). Another study isolated a strain of *Colletotrichum gloeosporioides* from *Huperzia serrata* that could produce Huperzine A (HupA), which found that lysine decarboxylase and copper amine oxidase are involved in the biosynthesis of HupA in *C. gloeosporioides* (Zhang et al., 2017). Subsequently, endophytic fungi were screened in a similar way as bioactive compounds of *S. alopecuroides* seeds. An endophytic *Alternaria that can product suspected* SR was initially found, numbered DSD201, and the concentration of the compound in the mycelium extract was 7.96 μg/mL (Supplementary Figure 4). However, it is not clear whether the other endophytic *Alternaria* or fungi species could produce similar bioactive compounds as their host, which will be the next steps in the future. This study sheds new light on the mechanism of QAs produced by *S. alopecuroides*.

Although the gene functions and interaction mechanisms of endophytes are unclear, we can gain meaningful information from correlation studies of endophyte diversity and stoichiometry. Based on Spearman correlation analysis and RDA, we found that the endophytic fungal community and dominant population of *S. alopecuroides* seeds were correlated with the main effective compounds. The richer, more even, and more diverse the endophytic fungal community are, the lower amount of OMA, OSC, and the total QAs (Figures 5A,C). This indicates that specific endophytic fungi play a key role in the accumulation of bioactive compounds in the internal environment of *S. alopecuroides* seeds. Through amplicon sequencing and PICRUSt functional prediction, Chen et al. (2018) found that the core microbiota of the medicinal plant *Salvia miltiorrhiza* seeds is a reservoir of beneficial microorganisms rich in secondary metabolism. Another study found that the secondary metabolites of *Rheum palmatum* were positively correlated with endophytic fungal diversity and abundance (Chen et al., 2021). This is contrary to the findings of this study, which may be due to plant specificity. Further research found that the endophytic fungus *Alternaria* of *S. alopecuroides* was positively correlated with the amount of OMA, OSC, and the total QAs, indicating that *Alternaria* promoted the production of QAs in the host (Figures 5B,D). Cui et al. (2021) reported the diversity of the endophytic fungal community of *Huperzia serrata* and analyzed its correlation with the production of HupA by host plants. There are 11 genera of endophytic fungi which were significantly positively correlated with the content of HupA in *Huperzia serrata*. In addition, the composition of endophytic fungi in *Cynomorium* is closely related to the accumulation of bioactive compounds in the host, including OTU1605, OTU2348, and OTU2595, which are highly correlated with differential metabolites (Cui et al., 2019). In the pot experiments of *Astragalus* and *Oxytropis*, plants with an SW concentration greater than 0.01% were marked as chemotype I, and a large number of *Undiflum* colonization was present, while an SW less than 0.01% was marked as chemical type II and was associated with a lower level of *Undifilum* colonization. Adding *Undifilum* to chemical type II increased the SW content to a level that was comparable to chemical type I (Grum et al., 2012). These studies demonstrated a potential correlation between specific endophytic fungi and host metabolites. That said, it is difficult to determine plant–microbe interactions in nature as both the species and number of fungi associated with each plant as well as the subsequent metabolite biotransformation is nearly incomprehensible.

## Conclusion

We explored the endophytic fungi in *S. alopecuroides* seeds by high-throughput sequencing of the ITS region of fungi. In this study, there were significant differences in the community diversity of endophytic fungi in *S. alopecuroides* in different areas. *Alternaria*, *Cladosporium*, *Filobasidium*, and an unidentified Ascomycota were the core-shared endophytes. Especially, it was found that *Alternaria* in *S. alopecuroides* seeds has the potential to promote the accumulation of bioactive compounds in the host.

## Data availability statement

The datasets presented in this study can be found in online repositories. The names of the repository/repositories and accession number(s) can be found below: https://www.ncbi.nlm.nih.gov/genbank/, SRP373295.

## Author contributions

MJ performed the experiments, analyzed the data, and wrote the manuscript. QZ contributed to the experiments and data interpretation and critically reviewed the manuscript. RW, SY, ZL, and PL collected samples, supervised the experiments, and validated the data. PG conceived the study, supervised the experiments and the data analysis, and wrote and critically reviewed the manuscript. All authors contributed to the article and approved the submitted version.

## Footnotes

1
https://unite.ut.ee/

2
http://drive5.com/uparse/
