# Supplementary material for: Correlation in endophytic fungi community diversity and bioactive compounds of Sophora alopecuroides
Source: Front Microbiol. 2022 Aug 31;13:955647. doi: 10.3389/fmicb.2022.955647 (PMC9475766; doi:10.3389/fmicb.2022.955647)
Supplement: Supplementary file 1 [file Data_Sheet_1.docx]

Supplementary Material

**Table S1.** Location information of *S. alopecuroides* sampling area

| **Location** | **Code No.** | **Longitude** | | **Latitude** | | **Altitude (m)** | |
| --- | --- | --- | --- | --- | --- | --- | --- |
| Manasi County, Xinjiang Autonomous Region | | MNSX | 86.193650 | | 44.276507 | | 526 |
| Heshuo County, Xinjiang Autonomous Region | | HSX | 86.863970 | | 42.268170 | | 1077 |
| Jiuquan City, Gansu Province | | JQS | 99.641654 | | 40.379171 | | 1166 |
| Ejin Banner, Inner Mongolia Autonomous Region | | EJN | 101.165051 | | 41.990427 | | 940 |
| Hainan Prefecture, Qinghai Province | | HNZ | 100.829048 | | 36.146046 | | 2569 |
| Longxi County, Gansu Province | | LXX | 104.640231 | | 35.008068 | | 2317 |
| Linhe District, Inner Mongolia Autonomous Region | | LHQ | 107.603225 | | 41.064038 | | 1033 |
| Hangjin Banner, Inner Mongolia Autonomous Region | | HJQ | 108.729340 | | 39.840230 | | 1391 |
| Huinong District, Ningxia Autonomous Region | | HNQ | 106.711450 | | 39.131930 | | 1091 |
| Yanchi County, Ningxia Autonomous Region | | YCX | 107.460358 | | 37.767742 | | 1336.4 |
| Longde County, Ningxia Autonomous Region | | LDX | 106.123320 | | 35.618120 | | 2096 |

**Table S2.** Standard curve in the detection of active compounds in *S. alopecuroides*

| **compound** | **linear regression*** | **R^2^** | **detection wavelength  (nm)** | **linear range  (****mg/mL)** |
| --- | --- | --- | --- | --- |
| OMA | y=1434.1x+25.131 | 0.9998 | 205 | 0.078~10.000 |
| OSC | y=1812.7x+62.971 | 0.9993 | 205 | 0.040~5.000 |
| SC | y=1449.7x+28.963 | 0.9996 | 205 | 0.200~2.500 |
| MA | y=1240.5x+9.9532 | 0.9995 | 205 | 0.005~0.600 |

Note: *: The x value is the concentration of the analyte (mg/mL), and the y value is the peak area of the tested compound.

**Table S3.** Annotations of 7 OTU species of endophytic fungi in the seed core-shared of *S. alopecuroides*

| **OTU ID** | **Phylum** | **Class** | **Order** | **Family** | **Genus** |
| --- | --- | --- | --- | --- | --- |
| OTU168 | Ascomycota | Dothideomycetes | Cladosporiales | Cladosporiaceae | *Cladosporium* |
| OTU119 | Ascomycota | Dothideomycetes | Cladosporiales | Cladosporiaceae | *Cladosporium* |
| OTU110 | Ascomycota | Dothideomycetes | Cladosporiales | Cladosporiaceae | *Cladosporium* |
| OTU196 | Ascomycota | unclassified Ascomycota | unclassified Ascomycota | unclassified Ascomycota | unclassified Ascomycota |
| OTU217 | Basidiomycota | Tremellomycetes | Filobasidiales | Filobasidiaceae | *Filobasidium* |
| OTU51 | Ascomycota | Dothideomycetes | Pleosporales | Pleosporaceae | *Alternaria* |
| OTU14 | Basidiomycota | Tremellomycetes | Filobasidiales | Filobasidiaceae | *Filobasidium* |

**Figure S1.** Rarefaction curve based on sobs index of endophyte fungi for 33 *S. alopecuroides* samples

**Figure S2.** Construction of core-shared fungal phylogenetic tree based on neighbor-joining method. Bootstrap support values are shown at the nodes. The tree was rooted to *Trypethelium* sp. (DQ782839) (Huang et al., 2009)

**Figure S3.** High-performance liquid chromatograms (A and B) and the individual mass spectrum (C~L) of the active compounds of the five alkaloids standard and representative sample. The blue, and red colours represent the alkaloids standard and representative sample, respectively. 1: matrine (MA), 2: sophocarpine and sophoridine (SC+SR), 3: oxysophocarpine (OSC), 4: oxymatrine (OMA).

**Figure S4.** Endophytic fungus DSD201 strain from *S. alopecuroides*, A, Spore characteristics, B, Colony morphology C, Sophoridine standard liquid chromatogram, D, DSD201 liquid chromatogram.
